# *In vitro* characterization of a novel resin-based restorative material containing alkaline fillers

**DOI:** 10.1590/1678-7757-2023-0219

**Published:** 2024-01-15

**Authors:** Matias Mederos, Elisa de León, Andrés García, Carlos Enrique Cuevas-Suárez, Juan Carlos Hernández-Cabanillas, José Alejandro Rivera-Gonzaga, Guillermo Grazioli

**Affiliations:** 1 Universidad de la Repúbica Facultad de Odontología Departamento de Odontología Preventiva y Restauradora Montevideo Uruguay Universidad de la Repúbica, Facultad de Odontología, Departamento de Odontología Preventiva y Restauradora, Area de Materiales Dentales, Montevideo, Uruguay; 2 Universidad Autónoma del Estado de Hidalgo Área académica de Odontología, Laboratorio de Materiales Dentales Pachuca México Universidad Autónoma del Estado de Hidalgo, Área académica de Odontología, Laboratorio de Materiales Dentales, Pachuca, México.; 3 Universidad Autónoma de Baja California Facultad de Ciencias de la Salud Tijuana México Universidad Autónoma de Baja California, Blvd Universitario, Facultad de Ciencias de la Salud, Tijuana, México.

**Keywords:** Polymers, Acid-base, Bioactive, Glass ionomer, Composite resins, Alkasite

## Abstract

**Objective:**

In this study, a comparative evaluation of the physicochemical properties of Cention N and other direct restorative materials was performed. Three restorative materials—a resin-modified glass ionomer (Fuji II LC), an alkasite-based resinous material (Cention N), and a resin composite (Tetric N Ceram)—were characterized in terms of degree of conversion, Knoop hardness number (KHN) ratio, flexural strength, elastic modulus, water sorption, water solubility, microshear bond strength to dentin, immediate microleakage, and radiopacity.

**Methodology:**

The microshear bond strength to dentin and microleakage of Cention N were evaluated with and without the application of an adhesive system (Tetric N Bond Universal). A one-way ANOVA test was used to analyze the data in terms of degree of conversion, KHN ratio, water sorption, water solubility, microshear bond strength to dentin, and radiopacity. A two-way ANOVA test (carried out considering the material type and ethanol aging as factors) was used to analyze the data in terms of flexural strength and elastic modulus. The Kruskal–Wallis test was used to statistically analyze the data on microleakage. A significance level of α=0.05 was used for all tests.

**Results:**

Fuji II LC was found to have the highest degree of conversion, water sorption, and microleakage, as well as the lowest flexural strength. Cention N had the highest solubility; when used with an adhesive system, it achieved bond strength and microleakage similar to those of the Tetric N Ceram composite. Tetric N Ceram had the highest degree of conversion, KHN ratio, and radiopacity. Conclusion: The properties of Cention N validate its efficacy as an alternative direct restorative material when used in conjunction with an adhesive system.

## Introduction

Glass ionomer cements (GICs) are valued for their ease of handling, fluoride release, specific adhesion to dental structures, and thermal expansion coefficient, which is similar to that of natural teeth.^1^ However, GICs have weak mechanical properties and are unsuitable for areas subject to stress.^2^ In contrast, resin-based composites (RBCs) offer aesthetically pleasing preservation of dental tissue, as they have micromechanical adhesion, low solubility, and strong mechanical properties. However, RBCs are sensitive to humidity and different placement techniques, and can lead to issues such as microleakage and secondary caries.^3^

Due to the fact that none of these materials fully meet the ideal requirements for restorative materials, a novel solution known as Cention N (Ivoclar; Schaan, Liechtenstein) has entered the market. Cention N is an esthetic (metal-free) and alkaline material categorized as an “alkasite”^4^ based on urethane dimethacrylate (UDMA) monomer. This material falls within the resin composite class and is enriched with alkaline glass fillers that promote the release of fluoride ions, hydroxyl groups, and calcium ions that neutralize acid around restorations and facilitate dental structure remineralization.^4^ Initially introduced as an amalgam substitute, this material has physical properties similar to those of amalgam and bioactive characteristics of GICs.^5^ According to the manufacturer, Cention N comprises dimethacrylates and light/chemical initiators. Consequently, Cention N can achieve high polymerization depth throughout a sample and can be applied to cavities using a monoincremental technique, with or without adhesive use.^6^

Recently, several studies have been conducted to assess the clinical performance of Cention N, investigating its effectiveness in restoring Class I, Class II, and Class V preparations in permanent and deciduous teeth.^7–9^ The results of these clinical trials have demonstrated that Cention N produces outcomes that are clinically acceptable in terms of retention, postoperative sensitivity, and secondary caries, comparable with those achieved usingresin composite materials. Additionally, Cention N has better clinical performance than glass ionomer cement. Notably, in most clinical trials, Cention N is used with an adhesive system. Based on these findings, Cention N appears to be a promising alternative for restoring occlusal caries lesions in both permanent and deciduous molars and is increasingly considered preferable to traditional glass ionomer cement. Despite these favorable results, the follow-up period of the abovementioned studies only extends up to 12 months, which reinforces the need to explore the properties of this material in depth.

As this novel material is recommended for restoring Class I and Class II cavities, characterization studies are needed to comprehensively understand its performance. Thus, the objective of this study was to comparatively evaluate the physicochemical properties of Cention N and of other direct restorative materials with similar clinical indications. The null hypothesis to be tested is that there are no differences among the properties of the evaluated materials.

## Methodology

### Study design

**Figure 1:**
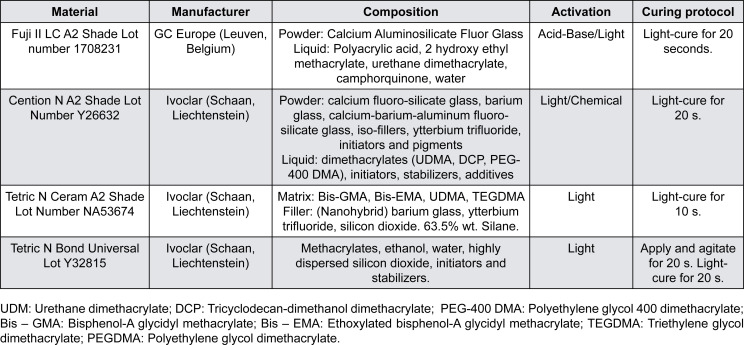
Technical profiles of the materials evaluated

An *in vitro* study was performed on three restorative materials: Fuji II LC, a resin-modified glass ionomer; Cention N, an alkasite-based resinous material; and Tetric N Ceram, a resin-based composite (Figure 1). The materials were characterized in terms of degree of conversion, Knoop hardness number (KHN) ratio, flexural strength (FS), elastic modulus, water sorption and solubility, microshear bond strength (mSBS) to dentin, immediate microleakage, and radiopacity. For Cention N, only the chemical + light activation mode was tested. Additionally, for this material, the mSBS test was conducted with or without applying an adhesive system (Tetric N Bond Universal), as specified by the manufacturer. The mSBS and microleakage tests were performed on extracted human third molars, following approval from the Ethical Review Board of the School of Medical Sciences, Autonomous University of Hidalgo State (Protocol CEEI-022-2021).

The primary response variable was mSBS (*n*=10). The sample size was estimated based on a previous study^10^ that evaluated the bond strength to dentin of Cention N, employing a comparative study design with four independent groups, a minimum detectable difference in means of 2.95, a standard deviation of 0.68, a power of 0.8, and an α=0.05. The sample size was calculated using software (SigmaPlot 12.0; Systat Software, Inc). Secondary response variables relating to the characterization of Cention N included FS and elastic modulus (*n*=10),^11^ degree of conversion (*n*=3),^12^ KHN ratio (*n*=5),^13^ water sorption and solubility (*n*=10),^14^ microleakage (*n*=10),^11^ and radiopacity (*n*=5).^15^ Randomization was achieved using a random number generator (Research Randomizer 4.0; Geoffrey C. Urbaniak and Scott Plous). A blinded operator performed all the physical and chemical tests conducted to analyze the chosen variables.

### Degree of conversion (DC)

The degree of conversion of the activated test materials was evaluated using a Fourier transform infrared spectrometer (Model IR-Prestige 21, Shimadzu Corporation, Japan) equipped with an attenuated total reflectance device composed of a horizontal ZnSe crystal with a 45° mirror angle (PIKE Technologies, Madison, WI, USA). A light-emitting diode (LED) light-curing unit (Optilight Max, Gnatus, Sao Paulo, Brazil) was attatched to a support coupled to the spectrometer in order to maintain a distance of 5 mm between the fiber tip and the sample during light activation. The irradiation values (1000 mW/cm^2^) were measured using a digital power meter (Bluephase meter, Ivoclar; Schaan, Liechtenstein).

The infrared spectra of the uncured and cured samples were obtained *(n*=3). Real-time measurements were conducted using attenuated total reflection. The height of the aliphatic C=C absorption peak observed at 1638 cm^−1^ and the carbonyl C=O absorption peak located at 1717 cm^−1^ was determined from each spectrum. The aromatic carbonyl C=O was used as an internal reference. Double bond conversion was determined using a previously described method.^16^

### KHN ratio

The cross-linking density of the materials was indirectly determined by measuring the microhardness of the test materials before and after softening in ethanol.^13^ For each material, five specimens (*n*=3; 5 mm × 2 mm × 2 mm) were built, embedded in acrylic resin, and polished with a metallographic polisher (PX300V2, LECO, Michigan, United States) using 600, 800, and 1200 grit sandpaper. The specimens were then dried at 37°C and stored for 24 h. Microhardness tests were performed on these specimens, with five indentations (each produced by applying a load of 500 g for 15 s) placed 100 μm apart. Assessments were conducted using a digital microhardness tester (HMV 2; Shimadzu, Tokyo, Japan). The microhardness was measured at three locations near the center of each specimen, and the resulting mean value was recorded as the initial KHN (the dry KHN). The specimens were then immersed in pure ethanol for 24 h, and the microhardness was measured again (the wet KHN). The ratio between the wet and dry KHNs (%) was used to indirectly measure the cross-linking density.

### FS and elastic modulus (EM)

The FS and elastic modulus of the polymers were measured using a mini-flexural three-point bending test. Customized stainless steel molds were used to fabricate ten bar-shaped specimens (dimensions: 10 mm × 2 mm × 2 mm) for each material group. Each restorative material was placed into a mold, which in turn was put on top of an acetate strip. The top and bottom surfaces of the specimens were then activated via light, with two irradiations of 20 s on each side, using the aforementioned LED light-curing unit (Optilight Max, Gnatus, São Paulo, Brazil). The polymerized specimens were removed from the molds and stored in distilled water at 37°C in the dark for 24 h. Five specimens from each group were subjected to a three-point bending test on a mechanical testing machine, MTS SANS CMT 2000 5Kn (MTS Systems Corporation; Shanghai, China), and measured using a digital caliper (Mitutoyo; Kawasaki, Kanagawa, Japan). The other five specimens in the group underwent an accelerated aging protocol in 100% ethanol for 7 days (*n* = 5). The load was centrally applied to the bar-shaped specimen at a cross-head speed of 0.5 mm/min until failure. The FS (σ) and elastic modulus (E) were calculated using the following equations:


$$\sigma =\frac{3Fl}{2bh^{2}}E=\frac{F_{1}l^{3}}{4bh^{3}d}$$

in which σ is the FS (MPa); *F* is the maximum load (N) exerted on the specimens at the point of fracture; *l* is the distance between the supports (10 mm); *b* is the width of the specimens (2 mm); *h* is the height of the specimens (2 mm); *E* is the elastic modulus (MPa) of the specimens; F1 is the force registered when the deformation stops being directly proportional to the force (N); d is the deflection corresponding to the load F1 (mm).

### Water sorption and solubility (WS/SL)

Ten specimens of each material were fabricated according to the ISO 4049 standard.^17^ Cylindrical specimens were polymerized in Teflon molds (with a 4-mm diameter and 1-mm thickness) and dry stored at 37°C. The specimens were weighed every 24 hours until they reached a constant mass (the mass loss of each specimen was not more than 0.1 mg at any time they were weighed), using an analytical digital balance (ME204, Mettler Toledo, Columbus, Ohio, USA) with an accuracy of 0.01 mg. Then, the thickness and diameter of the specimens were measured in order to calculate their volume (V). The specimens were individually immersed in distilled water and stored at 37°C. After seven days, the surface water of the specimens was removed, and the mass of each specimen was recorded again. The specimens were dry stored at 37°C and reweighed until they reached a constant mass. The water sorption and solubility of the specimens were calculated using the following equations:


$$W_{sp}=\frac{m_{2}-m_{3}}{V\text{​}}W_{sl}=\frac{m_{1}-m_{3}}{V}$$

in which *W*_sp_ is the water sorption value in μg, and *W*_sl_ is the specimen solubility in micrograms per cubic millimeter.

### mSBS

Human third molars were obtained for this study following ethical approval from the Ethical Review Board affiliated with the Health Sciences Institute at the Autonomous University of Hidalgo State under protocol CEEI-032-2019. A trimmer was used to wear down the occlusal surfaces of forty molars (*n* = 40) under copious water irrigation until a homogeneous dentin surface was exposed, avoiding pulp exposure. The molars were then placed in polyvinyl chloride (PVC) plastic tubes with a self-curing transparent acrylic resin in order to produce manipulable blocks for each specimen, leaving the dentin surface exposed and not covering the PVC tube. In order to be standardized, the exposed dentin surfaces of the specimens within the PVC tubes were polished using a metallographic polisher (PX300V2, LECO, Michigan, United States) at 200 rpm for 30 s using 600-grit wet sandpaper under irrigation. The dentin specimens were randomly divided into four groups according to the type of restorative material used: 1) a modified GIC, 2) Cention N without an adhesive, 3) Cention N with an adhesive, and 4) a resin composite with an adhesive. For groups 3 and 4, the universal adhesive Tetric N Bond was applied using the self-etch mode according to the manufacturer's instructions. Lastly, cylindrical elastomer molds (1 mm diameter by 1 mm height) were used to fabricate two blocks of the biomaterial of each specimen, manipulating this material according to the corresponding group. Ten teeth were treated per group, and the samples were stored at 37°C for 24 h before the microshear test.

The PVC tubes were fixed horizontally in a universal testing machine. An orthodontic wire was fixed to the upper arm of the machine to serve as a loop through which a button made of the test material could pass and pull laterally. The test was conducted using a 100-N load cell at a speed of 0.5 mm, in accordance with the ISO 11405 standard.^18^ The shear bond strength (SBS) was calculated in MPa.

### Microleakage

Forty intact third molars (*n* = 10) without carious lesions or prior restorations, which had been recently extracted for orthodontic purposes with written patient consent, were chosen for the *in vitro* study. After extraction, the teeth were cleaned of the remaining connective tissue and debris, rinsed with distilled water, and stored at room temperature. An experienced operator used a calibrated diamond bur under air-water cooling with a high-speed handpiece to create four Class V cavities with a depth of 4 mm (measured along the lateral wall), a width of 2 mm (measured along the pulpal wall), and a length of 3 mm (measured along the proximal wall) in each tooth. The margins of the cavities were finished using a fine diamond bur.

The Class V cavities were randomly restored using the following materials: 1) a modified GIC, 2) Cention N without an adhesive, 3) Cention N with an adhesive, and 4) a resin composite with an adhesive. For groups 3 and 4, the universal adhesive Tetric N Bond was applied using the self-etch mode according to the manufacturer's instructions. The restorations were finished using a fine-grit diamond bur, mounted in a water spray turbine, and polished using graded abrasive disks and rubbers. A total of 40 restorations were performed, ten in each group.

All tooth surfaces were coated with acetone-based nail varnish (Colorama, L'Oréal; Sao Paulo, Brazil), except for the restoration surface and a section of the tooth 1 mm from the tooth-restoration interface. The prepared samples were placed in distilled water at 37°C for 24 h and then in a 1% fuchsine solution at 37°C for 24 h, in accordance with the protocol established in the ISO 11405 standard.^18^ A low-speed diamond disk (LECO VC50, Michigan, United States) was used to bisect the samples in the middle of the cavity undergoing restoration, parallel to the occlusal surface. The depth of dye penetration into each point undergoing restoration was evaluated along the side walls of the teeth, measured using a stereoscope (ROSSBACH YZ-6, Rossbach, Mexico City, Mexico) at 10x magnification. Photographs of the restoration interface were obtained and analyzed based on a four-grade scale developed according to scales used in previous dental research studies. The grades in the scale were: 0—no dye penetration; 1—dye penetration into the enamel walls; 2—dye penetration into the walls up to the dentin; and 3—dye penetration down to the floor of the cavity.

### Radiopacity

The radiopacity of the materials was evaluated using five specimens per group, each measuring 6 mm in diameter and 1 mm in thickness. X-ray images were obtained using a digital system employing phosphorous plates (VistaScan; Dürr Dental GmbH & CO. KG, Bietigheim-Bissingen, Germany). The exposure time was set at 0.4 s, and the focus-film distance was 400 mm. The X-ray source (DabiAtlante model Spectro 70X) was operated with a tungsten anode at 70 kV and 8 mA. All images were obtained by simultaneously exposing an aluminum step-wedge to the specimens. The thickness of the aluminum step-wedge ranged from 0.5 to 5.0 mm in increments of 0.5 mm. The aluminum alloy used was composed of 99.12% Al, 0.47% Fe, 0.41% Mg, and <0.1% Cu (by mass) and met the ISO 6876 standards (Standardization 2001). The images were saved in TIFF format and analyzed using the Photoshop software (Adobe Systems Incorporated, San Jose, CA, USA). The means and standard deviations of the gray levels (pixel density) were measured in a standardized area of 1.5 mm^2^ in the images of the aluminum step-wedge and specimens.

### Statistical analysis

The sample size used for each test had a power of 0.8 and a significance level of 0.05. The normality of the data was verified using the Shapiro–Wilk test, and homoscedasticity was assessed using Levene's test. Data on the degree of conversion, KHN ratio, water sorption, water solubility, mSBS to dentin, and radiopacity were analyzed using one-way ANOVA and Tukey's post hoc tests. Data on FS and elastic modulus were analyzed with two-way ANOVA (using the material type and ethanol aging as factors) and Tukey's post hoc tests. Data on immediate microleakage were statistically analyzed using the Kruskal–Wallis test. A significance level of α = 0.05 was used for all tests.

## Results

Table 1 shows the degree of conversion of the evaluated materials. The statistical analysis showed that there were significant differences in the degrees of conversion of all the materials (*p*≤0.042). Overall, Fuji II LC had the highest degree of conversion and Tetric N Ceram had the lowest one. The tetric N Ceram specimens had the highest cross-linking densities (*p*<0.001), whereas the difference between Cention N and Fuji II LC was not statistically significant (*p*=0.082).

**Table 1:** Physical and chemical properties evaluated

Group	Degree of conversion (%)	KHN ratio (%)	Water sorption (μg/mm^3^)	Water solubility (μg/mm^3^)	Radiopacity (mmAl)
Tetric N Ceram	33.62 (1.34)^b^	61.87 (0.23)ᵃ	20.1 (4.3)^b^	3.0 (1.6)^b^	3.4 (0.2)ᵃ
Cention N	38.83 (2.70)^b^	48.08 (1.44)^b^	32.5 (4.8)^b^	21.6 (3.0)ᵃ	2.7 (0.2)^b^
Fuji II LC	77.18 (1.65)ᵃ	51.22 (2.3)^b^	173.4 (16.6)ᵃ	2.5 (1.4)^b^	2.6 (0.1)^b^

For each column, different superscript letters indicate the presence of statistically significant differences (p<0.05)

**Figure 2:**
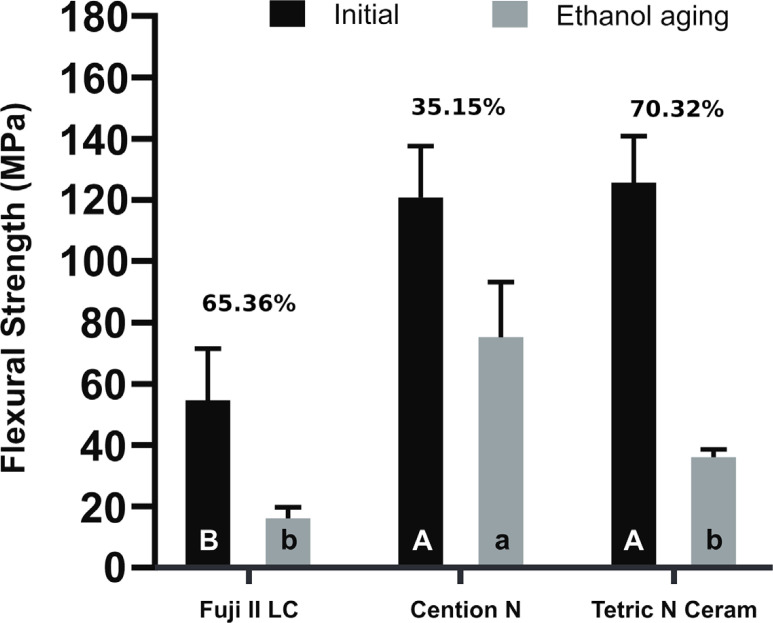
Flexural strength values before and after aging with ethanol. Equal superscript letters indicate the absence of statistically significant differences among the materials when evaluated immediately. Equal lower script letters indicate the absence of statistically significant differences among the materials when evaluated after undergoing ethanol aging. The differences in flexural strength after the immersion in ethanol were statistically significant for all materials

Figure 2 shows the FS of the materials before and after aging in ethanol. The material (*p*<0.001) and aging (*p*<0.001) factors significantly influenced FS, and there was a significant interaction between them (*p*<0.001). After 24 h of aging, the differences in the FS of Cention N and Tetric N Ceram were not statistically significant (*p*≥0.827) and were significantly higher than those in the Fuji II LC (*p*<0.001). After ethanol aging, Cention N achieved significantly higher FS values than Fuji II LC and Tetric N Ceram (*p*<.001). The decrease in FS after the immersion in ethanol was significant (*p*≤0.006) for all materials.

**Figure 3:**
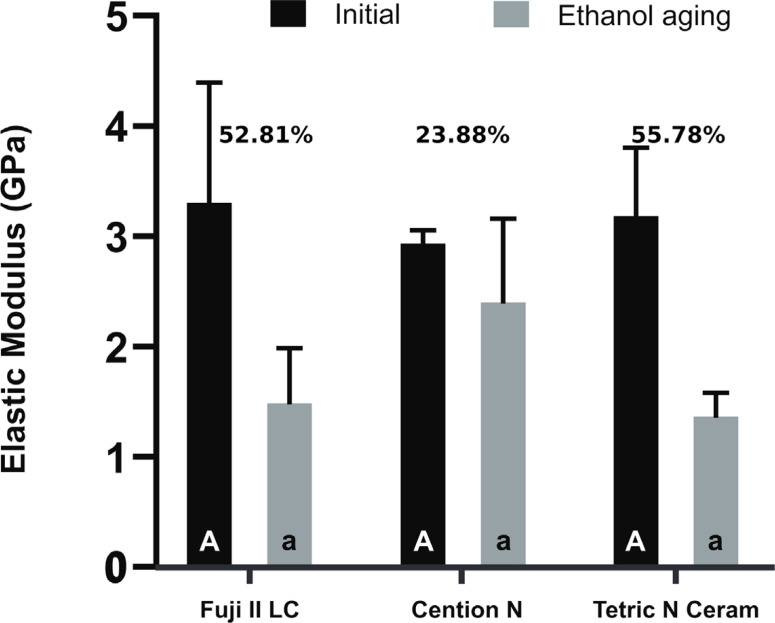
Elastic modulus values before and after aging with ethanol. Equal superscript letters indicate the absence of statistically significant differences among the materials when evaluated immediately. Equal lower script letters indicate the absence of statistically significant differences among the materials when evaluated after undergoing ethanol aging. All materials showed a statistically significant decrease in flexural strength after aging with ethanol

The statistical analysis showed that the aging factor significantly affected the elastic modulus (*p*<0.001, Figure 3), whereas the material type (*p*=0.514) and the interaction between the material and aging did not (*p*=0.068). After 24 hours of aging, no statistically significant differences were observed in elastic modulus between the materials (*p*≥0.582). After ethanol aging, the differences in elastic modulus between the materials were not statistically significant (*p*≥0.071), and all the materials showed a statistically significant decrease in the elastic modulus (p≤0.003).

Table 2 displays the results of the materials for water sorption, which was significantly higher for Fuji II LC than for the other materials (*p*<0.001). The difference between the water sorption values of Cention N and Tetric N Ceram was not statistically significant (*p*=0.177). Cention N was found to have the highest solubility (*p*>0.001), whereas the difference between the solubilities of Tetric N Ceram and Fuji II LC was not significant (*p*=0.838).

**Table 2:** Shear bond strength and microleakage values

Group	Microshear bond strength (MPa)	Microleakage
Tetric N Ceram	7.6 (3.3)ᵃ	0 (0-0)^b^
Cention N with adhesive	8.3 (2.9)ᵃ	0 (0-0.25)^b^
Cention N without adhesive	0.6 (1.0)^b^	3 (2.75-3)ᵃ
Fuji II LC	8.5 (3.4)ᵃ	2 (2-3)ᵃ

For each column, different superscript letters indicate the presence of statistically significant differences (p<0.05)

Table 2 shows the results for SBS and microleakage. The Cention N group without an adhesive had significantly lower SBS than the other materials (*p*<0.001), and the differences between the mSBS of the other materials were not statistically significant (*p*≥0.721). Cention N with an adhesive and Tetric N Ceram had the lowest immediate microleakage, and no significant differences were found between them.

Lastly, Table 1 shows that the most radiopaque material was Tetric N Ceram (*p*<0.001), whereas Cention N and Fuji II LC showed similar radiopacities (*p*=0.724).

## Discussion

In this *in vitro* study, we analyzed the mechanical, physical, and chemical properties of various esthetic direct restorative materials. Fuji II LC was found to have the highest degree of conversion, sorption, and microleakage, as well as the lowest FS. Conversely, Cention N had the highest solubility. Cention N applied without an adhesive had the lowest bond strength and the highest microleakage. In contrast, Tetric N Ceram specimens had the lowest degrees of conversion and the highest cross-linking densities and radiopacity. All materials showed similar elastic moduli, which led to the rejection of the null hypothesis initially proposed in this study.

The degree of conversion is a critical performance parameter correlated with other material properties.^19^ Herein, Fuji II LC showed the highest degree of conversion, followed by Cention N and Tetric N Ceram, as previously reported.^12^ The high degree of conversion of Fuji II LC can be attributed to its high content of hydroxyethyl methacrylate (HEMA). Monomers with a low glass transition temperature (Tg), such as HEMA, exhibit a higher degree of conversion than those with a high Tg, such as bisphenol glycidyl methacrylate (Bis-GMA) and triethylene glycidyl methacrylate.^12^ Furthermore, HEMA is a monofunctional monomer that forms linear polymers with a high degree of conversion.^20^ In contrast, Cention N contains novel photoinitiators such as Ivocerin, which allow it to have a higher degree of conversion than materials with conventional photoinitiators.^21^ The presence of these photoinitiators, along with that of polyethylene glycol dimethacrylate (PEGDMA), a monomer containing numerous heteroatoms such as oxygen, confers molecular flexibility and may explain the slightly elevated degree of conversion observed for Cention N.^22^

Tetric N Ceram showed the highest KHN ratio, due to the presence of bifunctional monomers such as Bis-GMA, which form stable cross-linked networks.^23^ In contrast, Cention N contains PEGDMA, a hygroscopic monomer highly susceptible to degradation, which leads to bond breaking and a less stable polymeric network with a reduced KHN ratio.^22^ Conversely, Fuji II LC showed a low KHN ratio despite having a high degree of conversion, which could be attributed to the linear polymers generated from HEMA, which are highly prone to hydrolytic degradation.^24^

Tetric N Ceram had an immediate FS similar to that of Cention N, whereas the immediate FS of Fuji II LC was statistically significantly lower than that of the two aforementioned materials, which is in line with observations made in another study.^6^ This difference in strength can be attributed to the fact that cross-linked polymer networks with bulky substituent groups can be found in Cention N and Tetric N Ceram, whereas Fuji II LC lacks rigid substituent functional groups in its polymer network.^25^ Both Cention N and Tetric N Ceram showed immediate FSs that complied with the specifications outlined in ISO 4049.^17^ In contrast, the immediate FS of Fuji II LC met the requirements of the ISO 9917-2 standard for water-based cements.^26^

No statistically significant differences were found among the elastic moduli of the tested materials. The elastic modulus of a composite depends on various features of the material, including the characteristics of the polymer matrix and the filler content.^27^ Furthermore, this property is directly related to the correct interaction between the organic and inorganic components. Specifically for resin-based composite materials, the silanization process of the inorganic filler is crucial for ensuring the proper transmission of forces between the organic and inorganic phases within the material.^28^ In Fuji II LC, the inorganic particles form a chemical bond with the organic matrix. Meanwhile, Tetric N Ceram and Cention N contain silanized filler particles. Overall, as specified by the manufacturers of the materials tested in this study, a correct interaction between the organic and inorganic phases is expected, which explains the similar elastic modulus observed in all the materials.^29^

The flexural properties of the materials were assessed after they underwent seven days of aging in 100% ethanol, which generated a decreased FS and elastic modulus in all materials, which is consistent with the findings of another study.^30^ The statistical analysis indicated that Cention N showed higher FS after the aging process compared with Tetric N Ceram and Fuji II LC, which suggests that Cention N has greater resistance to degradation. These results can be explained by the different monomers in the organic matrices of Cention N and Tetric N Ceram. Conversely, the low FS of Fuji II LC may be attributed to a low-molecular-weight HEMA monomer within its polymeric matrix.^31^

Fuji II LC showed significantly higher water sorption than Tetric N Ceram and Cention N, and no statistically significant differences were observed between the water sorption values of the latter two materials, which is consistent with previous reports.^14,32^ The water sorption values of Cention N and Tetric N Ceram complied with the maximum sorption limit of 40 μg/mm^3^ specified in the ISO 4049 standard.^17^ However, ISO 9917-2:2009 does not specify which sorption values water-based cements should achieve.^26^ The higher sorption of Fuji II LC may be attributed to the internal pores formed during the manual mixing of materials and the presence of highly hydrophilic HEMA.^33^ In contrast, Cention N contains the hydrophobic UDMA monomer, which makes it have lower sorption than Fuji II LC.^22^ Interestingly, the presence of the hydrophilic PEGDMA monomer in Cention N did not significantly affect its aqueous sorption.

Cention N was found to have significantly higher solubility than Fuji II LC and Tetric N Ceram. The measured solubility of Tetric N Ceram met the maximum specified value of 7.5 μg/mm^3^ outlined in the ISO 4049:2009 standard.^17^ The greater solubility of Cention N compared with the other two materials can be attributed to the fact that it contains alkaline fillers with a strong affinity for water.^32^ However, ISO 9917-2:2009 does not provide specific solubility values for water-based cements such as Fuji II LC.

No significant differences in bond strength to dentin were observed between Cention N used with Tetric N Bond Universal, Fuji II LC, and with Tetric N Ceram, which is in line with a previous report.^34^ The absence of differences between the bond strength of the Cention N and Tetric N Ceram groups can be attributed to the fact that the same adhesive system was used with them (Tetric N Bond Universal). The adhesive performance of Fuji II LC, for which an adhesive system was not used, may be attributed to the chemical adhesion and micromechanical retention induced by HEMA penetration into the dentinal tubules, as Fuji II LC is a hybrid material.^35^ Lastly, Cention N without an adhesive was found to have a statistically lower bond strength than the other materials, which may have resulted from the absence of any components enabling the establishment of a bond with dentin.^36^

The lowest microleakage values were observed in cavities restored with Tetric N Ceram and Cention N used with Tetric N Bond Universal, with no significant differences between these results, which is in line with findings from another report.^11^ This could be attributed to the adhesive system rather than the restorative material, as a well-functioning adhesive system can effectively seal a restoration.^37^ When Cention N was used without an adhesive, the highest microleakage values were observed. This could be due to the fact that it did not bond to the dental substrate, which, combined with the polymerization shrinkage of the material, increased the number of gaps between the restoration point and the tooth.^38^ Fuji II LC specimens were found to have bond strengths similar to those of Cention N specimens with an adhesive and Tetric N Ceram specimens, but had microleakage comparable to that of Cention N specimens without an adhesive. Although no direct correlation between adhesive strength and microleakage has yet been reported,^39^ the high microleakage observed for Fuji II LC could be attributed to the significant degradation of HEMA combined with its hydrophilicity.^40^

The radiopacity of the analyzed materials complied with the ISO 4049 requirement of a radiopacity ≥2 mm for Al.^17^ No significant difference was found between the radiopacities of Cention N and Fuji II LC, possibly due to the similarity in the properties and contents of their inorganic matrices.^41^ In contrast, Tetric N Ceram was found to have higher radiopacity than Cention N and Fuji II LC, which can be attributed to its higher ytterbium fluoride content.^42^

One limitation of this study is its *in vitro* design. In addition, resinous and ionomeric materials available in the market were not included in this study and could exhibit different behaviors from those reported here. As some materials contain different photoinitiators, it could be interesting to evaluate their properties upon polymerization with a polywave lamp. Lastly, the bond strength of the tested materials was only evaluated after they underwent 24 h of aging, although it is important to evaluate the long-term behavior of materials. Despite these limitations, to the best of our knowledge, no other studies in the literature have comparatively analyzed as many properties of this novel Cention N material as our study.

## Conclusion

Our study confirms that Cention N meets the international standards for flexural properties, water sorption, and radiopacity. When used in conjunction with an adhesive system, this material exhibits bond strength and microleakage comparable to those of traditional resin composites, which reinforces the importance of this combination. Moreover, the degree of conversion and KHN ratio of the alkasite material are similar to those of its counterparts. However, concerns regarding the solubility of the material still arise, which makes it necessary to conduct further investigations, particularly via rigorous clinical trials, to validate its clinical performance and long-term suitability as a dental restorative material.

## References

[1] Sidhu SK, Nicholson JW (2016). A review of glass-ionomer cements for clinical dentistry. J Funct Biomater.

[2] Menezes-Silva R, Cabral RN, Pascotto RC, Borges AFS, Martins CC, Navarro MF (2019). Mechanical and optical properties of conventional restorative glass-ionomer cements-a systematic review. J Appl Oral Sci.

[3] Veiga AM, Cunha AC, Ferreira DM, Fidalgo TK, Chianca TK, Reis KR (2016). Longevity of direct and indirect resin composite restorations in permanent posterior teeth: a systematic review and meta-analysis. J Dent.

[4] Meshram P, Meshram V, Palve D, Patil S, Gade V, Raut A. (2019). Comparative evaluation of microleakage around Class V cavities restored with alkasite restorative material with and without bonding agent and flowable composite resin: an *in vitro* study. Indian J Dent Res.

[5] Tiskaya M, Al-Eesa N, Wong F, Hill R. (2019). Characterization of the bioactivity of two commercial composites. Dent Mater.

[6] Iftikhar N, Srivastava B, Gupta N, Ghambir N. (2019). A comparative evaluation of mechanical properties of four different restorative materials: an *in vitro* study. Int J Clin Pediatr Dent.

[7] Sharma H, Suprabha B, Shenoy R, Rao A, Kotian H. (2023). Clinical effectiveness of alkasite versus nanofilled resin composite in the restoration of occlusal carious lesions in permanent molar teeth of children: a randomized clinical trial. Eur Arch Paediatr Dent.

[8] Oz FD, Meral E, Gurcan S. (2023). Clinical performance of an alkasite-based bioactive restorative in class II cavities: a randomized clinical trial. J Appl Oral Sci.

[9] Derchi G, Marchio V, Giuca MR, Lardani L. (2022). Clinical performance of centiontm alkasite restorative material vs. glass ionomer cement used in deciduous teeth: one-year evaluation. Appl Sci.

[10] Verma V, Mathur S, Sachdev V, Singh D. (2020). Evaluation of compressive strength, shear bond strength, and microhardness values of glass-ionomer cement Type IX and Cention N. J Conserv Dent.

[11] Sujith R, Yadav TG, Pitalia D, Babaji P, Apoorva K, Sharma A. (2020). Comparative evaluation of mechanical and microleakage properties of Cention-N, composite, and glass ionomer cement restorative materials. J Contemp Dent Pr.

[12] Panpisut P, Toneluck A. (2020). Monomer conversion, dimensional stability, biaxial flexural strength, and fluoride release of resin-based restorative material containing alkaline fillers. Dent Mater J.

[13] Pérez-Mondragón AA, Cuevas-Suárez CE, González-López JA, Trejo-Carbajal N, Meléndez-Rodríguez M, Herrera-González AM (2020). Preparation and evaluation of a BisGMA-free dental composite resin based on a novel trimethacrylate monomer. Dent Mater.

[14] Nayak M, Shenoy V. (2019). Sorption and solubility of alkasite restorative material-an *in vitro* study. IOSR J Dent Med Sci.

[15] Balci M, Turkun L, oglu H, Guneri P, Ergucu Z. (2023). Radiopacity of posterior restorative materials: a comparative *in vitro* study. Oper Dent.

[16] Herrera-González AM, Caldera-Villalobos M, Pérez-Mondragón AA, Cuevas-Suárez CE, González-López JA (2019). Analysis of double bond conversion of photopolymerizable monomers by FTIR-ATR spectroscopy. J Chem Educ.

[17] International Organization for Standardization (2019). ISO 4049:2019: polymer based materials.

[18] International Organization for Standardization (2003). ISO/TS 11405:2003: dental materials — testing of adhesion to tooth structure.

[19] Cuevas-Suárez CE, Pimentel-García B, Rivera-Gonzaga A, Álvarez-Gayosso C, Ancona-Meza AL, Grazioli G (2018). Examining the effect of radiant exposure on commercial photopolimerizable dental resin composites. Dent J.

[20] Collares FM, Ogliari FA, Zanchi CH, Petzhold CL, Piva E, Samuel S. (2011). Influence of 2-hydroxyethyl methacrylate concentration on polymer network of adhesive resin. J Adhes Dent.

[21] Sanay B, Strehmel B, Strehmel V. (2020). Photoinitiated polymerization of methacrylates comprising phenyl moieties. J Polym Sci.

[22] Fugolin AP, Paula AB, Dobson A, Huynh V, Consani R, Ferracane JL (2020). Alternative monomer for BisGMA-free resin composites formulations. Dent Mater.

[23] Ogliari FA, Ely C, Zanchi CH, Fortes CB, Samuel SM, Demarco FF (2008). Influence of chain extender length of aromatic dimethacrylates on polymer network development. Dent Mater.

[24] Tauscher S, Angermann J, Catel Y, Moszner N. (2017). Evaluation of alternative monomers to HEMA for dental applications. Dent Mater.

[25] Bakkal M, Yılmaz B, Durmus A, Durmus Z, Ozalp S. (2019). Polymerization characteristics of colored compomers cured with different LED units. J Appl Biomater Funct Mater.

[26] International Organization for Standardization (2017). ISO 9917-2:2017 Dentist- water-based cements — Part 2: Resin-modified cements.

[27] Masouras K, Silikas N, Watts DC. (2008). Correlation of filler content and elastic properties of resin-composites. Dent Mater.

[28] Yoshida Y, Shirai K, Nakayama Y, Itoh M, Okazaki M, Shintani H (2002). Improved filler-matrix coupling in resin composites. J Dent Res.

[29] Ilie N. (2018). Comparative Effect of self- or dual-curing on polymerization kinetics and mechanical properties in a novel, dental-resin-based composite with alkaline filler. Materials (Basel).

[30] Witzel MF, Calheiros FC, Gonçalves F, Kawano Y, Braga RR (2005). Influence of photoactivation method on conversion, mechanical properties, degradation in ethanol and contraction stress of resin-based materials. J Dent.

[31] Carvalho AA, Moreira FC, Fonseca RB, Soares CJ, Franco EB, Souza JB (2012). Effect of light sources and curing mode techniques on sorption, solubility and biaxial flexural strength of a composite resin. J Appl Oral Sci.

[32] Müller JA, Rohr N, Fischer J. (2017). Evaluation of ISO 4049: water sorption and water solubility of resin cements. Eur J Oral Sci.

[33] Beriat NC, Nalbant D. (2009). Water absorption and HEMA release of resin-modified glass-ionomers. Eur J Dent.

[34] Awad MM, Alshehri T, Alqarni AM, Magdy NM, Alhalabi F, Alotaibi D (2020). Evaluation of the bond strength and cytotoxicity of alkasite restorative material. Appl Sci.

[35] Rusin RP, Agee K, Suchko M, Pashley DH. (2010). Effect of a new desensitizing material on human dentin permeability. Dent Mater.

[36] François P, Greenwall-Cohen J, Le Goff S, Ruscassier N, Attal JP, Dursun E. (2020). Shear bond strength and interfacial analysis of high-viscosity glass ionomer cement bonded to dentin with protocols including silver diammine fluoride. J Oral Sci.

[37] Kaczor–Wiankowska K, Lipa S, Krasowski M, Sokołowski J, Lewusz–Butkiewicz K, Nowicka A. (2020). Evaluation of gap formation at the composite resin–tooth interface after using universal adhesives: *in vitro* SEM study using the replica technique. Microsc Res Tech.

[38] Correia A, Andrade MR, Tribst JP, Borges AL, Caneppele TM (2020). Influence of bulk-fill restoration on polymerization shrinkage stress and marginal gap formation in Class V restorations. Oper Dent.

[39] León Cáceres ME, Mederos Gómez M, Cuevas-Suárez CE, Maglione García F, Grazioli Pita GS (2020). Estudio *in vitro* de la relación entre resistencia de unión a esmalte dental y microfiltración en resinas compuestas fotopolimerizables. Odontoestomatología.

[40] Bayrak S, Sen Tunc E, Tuloglu N. (2012). The effects of surface pretreatment on the microleakage of resin-modified glass-ionomer cement restorations. J Clin Pediatr Dent.

[41] Aoyagi Y, Takahashi H, Iwasaki N, Honda E, Kurabayashi T. (2005). Radiopacity of experimental composite resins containing radiopaque materials. Dent Mater J.

[42] Sabbagh J, Vreven J, Leloup G. (2004). Radiopacity of resin-based materials measured in film radiographs and storage phosphor plate (Digora). Oper Dent.

